# Controlling behaviours and technology‐facilitated abuse perpetrated by men receiving substance use treatment in England and Brazil: Prevalence and risk factors

**DOI:** 10.1111/dar.12521

**Published:** 2017-01-30

**Authors:** Gail Gilchrist, Martha Canfield, Polly Radcliffe, Ana Flavia Pires Lucas D'Oliveira

**Affiliations:** ^1^ National Addiction Centre, Institute of Psychiatry, Psychology and NeuroscienceKing's College LondonLondonUK; ^2^ Gender Violence and Health Research Group, Department of Preventive MedicineUniversity of Sao PauloSao PauloBrazil

**Keywords:** intimate partner violence, controlling behaviour, technology‐facilitated abuse, substance use treatment, cross‐sectional study

## Abstract

**Introduction and Aims:**

Controlling behaviours are highly prevalent forms of non‐physical intimate partner violence (IPV). The prevalence of perpetrating controlling behaviours and technology‐facilitated abuse (TFA) was compared by men receiving substance use treatment in England (n = 223) and Brazil (n = 280). Factors associated with perpetrating these behaviours towards their current/most recent partner and their association with other types of IPV were explored.

**Design and Methods:**

Secondary analysis from two cross‐sectional studies was performed. Data on socio‐demographic characteristics, infidelity, IPV perpetration and victimisation, adverse childhood experiences (ACE), attitudes towards gender relations and roles, substance use, depressive symptoms and anger expression were collected.

**Results:**

Sixty‐four percent (143/223) and 33% (73/223) of participants in England and 65% (184/280) and 20% (57/280) in Brazil reported controlling behaviours and TFA, respectively, during their current/most recent relationship. Excluding IPV victimisation from the multivariate models; perpetrating controlling behaviours was associated with a higher number of ACE, higher anger expression (England) and severe physical IPV perpetration (Brazil), and perpetrating TFA was associated with younger age. Including both IPV victimisation and perpetration in the multivariate models; perpetrating controlling behaviour was associated with experiencing a higher number of ACE, higher anger expression (England), emotional IPV victimisation (England) and experiencing controlling behaviour from a partner (England). The perpetration of TFA was associated with younger age and experiencing TFA from a partner.

**Conclusions:**

Technological progress provides opportunities for perpetrators to control and abuse their partners. Controlling behaviours and TFA should be addressed to reduce IPV perpetration by males in substance use treatment. [Gilchrist G, Canfield M,Radcliffe P, d'Oliveira AFPL. Controlling behaviours and technology‐facilitated abuse perpetrated by men receiving substance use treatment in England and Brazil: Prevalence and risk factors. *Drug Alcohol Rev* 2017;36:52–63]

## Introduction

Coercive control has been described as ‘*an ongoing pattern of domination by which male abusive partners primarily interweave repeated physical and sexual violence with intimidation, sexual degradation, isolation and control*’ [1, pp. 7]. Controlling behaviour is a highly prevalent form of non‐physical violence, and while it often co‐occurs with physical and sexual violence in intimate relationships 2, 3, different opinions exist over whether it is a constituent part of intimate partner violence (IPV) 4, 5, 6. Various IPV perpetrator typologies have been proposed classifying IPV on the ‘nature of the violence’ 7 or ‘the psychological profiles of perpetrators’ [8, in 9]. Controlling behaviour in the intimate terrorism typology (i.e. ‘a pattern of emotionally abusive intimidation, coercion, and control’ with or without physical violence) [7, pp.478–479] has been associated with the most severe physical assaults and is typically perpetrated by men 10, 11, 12. There remains a lack of consensus on what non‐violent acts should be included in the definition of IPV 13.

Several large population‐based surveys have examined the prevalence of controlling or coercive behaviours, albeit using differing methods; for example, in a recent UK study 9% of men and 21% of women reported ever experiencing non‐physical partner violence (emotional, financial) 14, 37% of men and 41% of women in a Swedish study had experienced isolating control from a partner in the past year (including restricting time spent with family/friends; wanting to know partner's whereabouts, suspicion or jealousy) 15 and 63% of women in a Nigerian study had experienced controlling behaviour from a partner (i.e. jealous if talks with men, accusations of unfaithfulness, does not permit her to meet friends, limits contact with family) 2. Behaviours that include control over a partner's access to resources, freedom of movement and decision making negatively affect the victim 16, 17 and it is argued can be equal to or more threating than physical or sexual assault 5, 13, 18.

A wide variety of technology is now being used to abuse partners, including email, text messaging, phone calls, social media and GPS tracking 19, 20, 21, 22. Studies suggest as many as 40–73% of US college students have experienced such cyber abuse in dating relationships, and that cyber abuse was associated with experiencing other forms of psychological, physical and sexual partner abuse 23, 24. In a recent study of female survivors of IPV in Australia, 78% reported receiving text messages, phone calls etc. in which they had been called names, harassed or ‘put down’, 56% reported that partners had used mobile technology to check their location, and 17% reported partners had used GPS technology to track them 22. The increasingly widespread use of mobile technologies 25 provides a means for perpetrators to easily and repeatedly control, harass, stalk and intimidate partners from a distance 22, 26.

To date, existing evidence concerning who is most likely to perpetrate controlling behaviours 27 and other forms of IPV is mainly derived from victims' experiences 28, 29, contributing to our limited understanding of whether controlling behaviours and technology‐facilitated abuse (TFA) have similar underlying risk factors and whether both behaviours are linked. More critically, research suggests that victims of cyber or technology stalking do not always perceive that they are experiencing abuse 22. Understanding the factors associated with controlling behaviours and TFA, and the role of TFA as a ‘course of conduct’ 30 could offer ways of improving the detection of abuse.

Previous research has shown that rates for IPV perpetration are higher among men receiving treatment for substance use than for men in the general population 31, 32, 33. While alcohol and drug use are widely accepted risk factors for IPV, younger age, negative childhood experiences, psychological problems and anger expression are also risk factors 34, 35. IPV prevalence is higher in low‐income than high‐income countries 36. Societies that support stronger ideologies of male dominance have elevated rates of IPV 37. A recent analysis of IPV prevalence studies from 44 countries found that women who accept wife beating as a man's right and who have a controlling partner are at a significantly higher risk of violence 36. While the prevalence rates of controlling behaviour and physical and sexual IPV vary across cultures and countries, the association between these behaviours persists 38, 39. Men who are physically violent towards their partners report higher rates of controlling behaviours than men who are not physically violent 39. Further research is required to answer how this varies across cultural settings and what role for example ‘machismo’ culture (a system of values and ideas that institutes, reinforces and legitimises men's domination over women) 40 may play.

Although there is growing recognition of controlling behaviours and TFA, the prevalence of these behaviours remains understudied, especially among high risk groups for other IPV perpetration, such as men receiving treatment for substance use. This study examined the prevalence of controlling behaviours and TFA by men receiving treatment for substance use in two distinct societies, England and Brazil (greater use of cocaine, greater gender inequality and higher prevalence of IPV and general violence in Brazil compared to England) 41, 42, 43, 44, 45, 46, 47 and describes the sociodemographic, psychological, and cultural factors associated with perpetrating controlling behaviour and TFA.

## Methods

### Design

A secondary analysis of two cross‐sectional studies (in England and Brazil) on the prevalence of IPV perpetration by men attending substance use treatment 48 was performed.

### Procedure

Potential participants were approached by researchers in waiting rooms of community substance use treatment services (six in São Paulo, three in London and three in south‐east England) and invited to participate in the study. Researchers explained the study verbally and gave all potential participants a participant information sheet. Informed consent was gained before the interview was conducted in a private room of the treatment facility. A convenience sample of 519 men aged 18 year or above were recruited. Further details on ethical approval, the services and recruitment procedure are reported earlier in this issue 48.

### Measurements

Translation of instruments into Portuguese is explained elsewhere 48.

#### Socio‐demographics

Participants' age, living arrangements, highest level of education attained, current employment status and whether the participant lived in the country of birth were recorded.

#### Infidelity

Participants reported whether they had a sexual relationship with another woman/man during their current/most recent relationship.

#### Controlling behaviour

Participants were asked whether it was generally true that they had perpetrated at least one of seven controlling behaviours towards their current/most recent partner (Table 1) 49.

**Table 1:** Differences in controlling behaviours and technology facilitated abuse by country

	Brazil (*N* = 280)	England (*N* = 223)	*P*	OR (95% CI)
*Controlling behaviours (7 items)*				
Try to keep her/him from seeing her/his friends	56 (20.0%)	20 (9.0%)	**<0.001**	0.39 (0.23, 0.68)
Try to restrict contact with her/his family of birth	19 (6.8%)	5 (2.2%)	**0.018**	0.31 (0.12, 0.86)
Insist on knowing where she/he is at all times	88 (31.5%)	53 (23.8%)	0.054	0.68 (0.45, 1.01)
Ignore her/him and treat her/him indifferently	57 (20.5%)	82 (36.8%)	**<0.001**	2.25 (1.51, 3.36)
Get angry if she/he speaks with another man	103 (36.9%)	53 (23.8%)	**0.002**	0.53 (0.36, 0.79)
Am often suspicious that she/he is unfaithful	86 (30.8%)	83 (37.2%)	0.132	1.33 (0.92, 1.93)
Expect her/him to ask your permission before seeking health care for herself/himself	23 (8.2%)	2 (0.9%)	**<0.001**	0.10 (0.02, 0.43)
At least one controlling behaviour perpetrated	184 (65.9%)	143 (64.1%)	0.670	0.92 (0.63, 1.33)
Number of controlling behaviour items [mean (SD)]	1.54 (1.57)	1.38 (0.09)	0.122	0.79 (0.65, 0.95)
*Technology facilitated abuse (4 items)*				
Use text messages, phone, etc. to call her/him names, harass her/him or put her/him down	22 (7.9%)	38 (17.1%)	**0.001**	2.42 (1.39, 4.23)
Use mobile technology to check where she/he is	15 (5.4%)	13 (5.8%)	0.818	1.09 (0.51, 2.35)
Check her/his text messages without her/his permission	42 (15.0%)	46 (20.6%)	0.099	1.47 (0.93, 2.34)
Threaten her/him via text, email or over social media	10 (3.6%)	15 (6.8%)	0.103	1.96 (0.86, 4.44)
Any technology facilitated abuse	57 (20.4%)	73 (32.7%)	**0.002**	1.90 (1.27, 2.85)
Number of technology facilitated abuse items [mean (SD)]	0.32 (0.75)	0.82 (0.07)	**0.014**	1.15 (0.73, 1.80)

CI, confidence interval; OR, odds ratio.

#### Technology‐facilitated abuse

TFA was considered to have taken place if participants reported it was generally true that they had conducted at least one of the four behaviours towards their current/most recent partner (Table 1) 50.

#### Intimate partner violence

Lifetime victimisation and perpetration of emotional, physical or sexual IPV were assessed using questions from the World Health Organization Multi‐country Study on women's health and domestic violence against women 49. Participants were also asked whether they had ever had a physical fight with a man.

#### Adverse childhood experiences

Ten adverse childhood experiences (ACE) were assessed: being looked after or adopted, neglect, parental death, separation/divorce, mother never/rarely at home, father never/rarely at home and being told you were weak or lazy; childhood sexual, physical abuse, witnessing inter‐parental violence 51, 52, 53. Mean adverse childhood experiences scores were calculated for respondents to all 10 experiences.

#### Substance use

The Alcohol Use Disorders Identification Test 54 assessed alcohol consumption and related problems in the previous year. A total score of ≥8 indicates hazardous drinking 55. Participants were asked whether they were currently receiving (or in the past) treatment for use of alcohol and/or drug/s. Participants' current/most recent partners' alcohol or drug use was also recorded.

#### Mental health and anger expression

The Primary Care Evaluation of Mental Disorders Patient Health Questionnaire 56 was administered to assess depressive symptoms. Participants are asked to indicate how often they have experienced each symptom in the past two weeks using a Likert Scale ranging from 0 (not at all) to 3 (nearly every day). A score of ≥10 determined probable major depressive disorder. Participants were also asked whether they had ever been told by a health professional that they had manic depressive illness or bipolar disorder.

The anger expression index was calculated from four sub‐scales from the State–Trait Anger Expression Inventory‐2 57. Scores range from 0 to 96, with higher scores indicating a higher tendency to express anger either externally towards other persons or objects or internally towards their self.

#### Attitudes

The 17 item Attitudes to Gender Relations Scale 38 assessed views on relations between men and women in society. Responses range from 1 (strongly disagree) to 5 (strongly agree). Scores ranged from 17 to 69 with higher scores representing higher support for gender equitable norms. The Attitudes Towards Gender Roles Scale 49 was also administered. Participants were asked agree or disagree with 16 statements about families and acceptable behaviour for men and women in the home. Scores ranged from 16 to 32, with lower scores representing more gender stereotyped attitudes towards gender roles.

### Analysis

Descriptive statistics were calculated. Differences were assessed using *t*‐tests for continuous data and χ^2^ tests for categorical data. Odds ratios (OR) and 95% confidence intervals (CI) were calculated using logistic regression. Table 1 presents the distribution of responses to the controlling behaviours and TFA items by country. Table 2 describes variables associated with controlling behaviour and TFA by country in univariate analysis. Variables with cell counts of >10 and *P* ≤ 0.2 in the univariate analyses were entered into separate backward stepwise logistic regression to ascertain variables associated with controlling behaviour and TFA, excluding (Table 3) and including (Table 4) other types of IPV victimisation for each country to allow the issue of gender symmetry in IPV victimisation to be considered 4.

**Table 2:** Univariate factors associated with perpetrating controlling behaviours and technology facilitated abuse: mean (SD) and number (%)

Brazil	Controlling behaviours				Technology facilitated abuse			
	No, *N* = 95	Yes, *N* = 184	*P*	OR (95% CI)	No, *N* = 223	Yes, *N* = 57	*P*	OR (95% CI)
*Demographics*								
Age [mean (SD)]	46.17 (11.26)	41.84 (11.16)	**0.002**	0.97 (0.94, 0.98)	45.06 (11.31)	36.65 (8.87)	**<0.001**	0.41 (0.25, 0.66)
Heterosexual	91 (95.8%)	179 (97.3%)	0.504	1.57 (0.41, 6.00)	214 (96.0%)	57 (100.0%)	0.123	‐
Live in the country of birth	94 (98.9%)	182 (98.9%)	0.979	0.97 (0.09, 10.81)	221 (99.1%)	56 (98.2%)	0.575	0.51 (0.45, 5.69)
No/primary schooling/left school without qualifications	61 (64.2%)	117 (63.9%)	0.964	0.99 (0.59, 1.66)	147 (66.2%)	31 (54.4%)	0.097	0.52 (0.29, 0.91)
Unemployed/receiving benefits	33 (34.7%)	91 (49.5%)	**0.019**	1.84 (1.10, 3.07)	98 (43.9%)	27 (47.4%)	0.643	1.15 (0.64, 2.06)
Homeless	2 (2.1%)	10 (5.5%)	0.191	2.69 (0.58, 12.52)	10 (4.5%)	2 (3.5%)	0.741	0.77 (0.16, 3.62)
*Intimate relationship*								
Infidelity	36 (38.3%)	97 (53.6%)	**0.016**	1.85 (1.12, 3.09)	98 (44.7%)	36 (63.2%)	**0.013**	2.12 (1.16, 3.86)
Believed that current/most recent partner has/had a problem with alcohol or drug use	25 (26.3%)	39 (21.2%)	0.335	0.75 (0.42, 1.34)	47 (21.1%)	17 (29.8%)	0.160	1.60 (0.83, 3.06)
*Substance use*								
In treatment for alcohol	85 (90.4%)	154 (83.7%)	0.126	0.54 (0.25, 1.20)	200 (90.1%)	40 (70.2%)	**<0.001**	0.26 (0.13, 0.53)
In treatment for drug	45 (47.4%)	113 (61.4%)	**0.025**	1.77 (1.07, 2.92)	116 (52.0%)	42 (73.7%)	**0.003**	2.58 (1.35, 4.92)
Hazardous drinking	66 (69.5%)	142 (77.2%)	0.162	1.49 (0.85, 2.59)	162 (72.6%)	46 (80.7%)	0.214	1.57 (0.77, 3.24)
AUDIT total score [mean (SD)]	17.09 (11.86)	19.97 (12.20)	0.061	1.02 (0.99, 1.04)	18.90 (12.25)	19.09 (11.82)	0.920	1.00 (0.98, 1.03)
*Anger [mean (SD)]*								
Anger Expression Index	32.37 (15.25)	42.28 (16.58)	**<0.001**	1.04 (1.02, 1.06)	36.62 (16.01)	47.82 (16.91)	**<0.001**	1.04 (1.02, 1.06)
*Violence*								
Victim of controlling behaviours	69 (72.6%)	173 (94.0%)	**<0.001**	5.93 (2.78, 12.65)	187 (83.9%)	56 (98.2%)	**0.020**	10.78 (1.45, 80.40)
Victim of TFA	24 (25.3%)	78 (42.4%)	**0.005**	2.90 (1.63, 5.16)	59 (26.5%)	44 (77.2%)	**0.001**	9.41 (4.74, 18.69)
IPV victimisation								
Emotional	56 (58.9%)	141 (76.6%)	**0.002**	2.28 (1.34, 3.89)	150 (67.3%)	48 (84.2%)	**0.012**	2.60 (1.21, 5.58)
Sexual	8 (8.4%)	39 (21.2%)	**0.007**	2.92 (1.31, 6.55)	30 (13.5%)	17 (29.8%)	**0.003**	2.73 (1.38, 5.43)
Moderate physical	13 (32.5%)	27 (24.8%)	0.345	0.68 (0.31, 1.51)	31 (29.0%)	9 (20.9%)	0.314	0.65 (0.28, 1.51)
Severe physical	27 (67.5%)	82 (75.2%)	0.345	1.46 (0.66, 3.23)	76 (71.0%)	34 (79.1%)	0.314	1.54 (0.66, 3.59)
*Violence*								
IPV perpetration								
Emotional	44 (46.3%)	126 (68.5%)	**<0.001**	2.52 (1.51, 4.19)	125 (56.1%)	46 (80.7%)	**0.001**	3.28 (1.61, 6.66)
Sexual	6 (6.3%)	23 (12.5%)	0.109	2.12 (0.83, 5.40)	17 (7.6%)	12 (21.1%)	**0.003**	3.23 (1.44, 7.23)
Moderate physical	10 (35.7%)	10 (12.2%)	0.005	0.25 (0.90, 0.69)	20 (24.4%)	1 (3.4%)	**0.013**	0.11 (0.15, 0.89)
Severe physical	18 (64.3%)	72 (87.8%)	**0.005**	4.00 (1.45, 11.06)	62 (75.6%)	28 (96.6%)	**0.013**	9.03 (1.15, 70.68)
Ever physical fight with a man	66 (70.2%)	151 (83.0%)	**0.014**	2.07 (1.15, 3.72)	170 (76.9%)	47 (83.9%)	0.256	1.57 (0.72, 3.41)
*Mental health*								
PHQ total score [mean (SD)]	8.42 (7.08)	11.66 (7.59)	**0.001**	1.06 (1.02, 1.10)	9.79 (7.42)	13.42 (7.51)	**0.001**	1.06 (1.02, 1.11)
Probable depressive disorder	33 (34.7%)	101 (54.9%)	**0.001**	2.29 (1.37, 3.82)	96 (43.0%)	38 (66.7%)	**0.001**	2.65 (1.44, 4.87)
ACE total score [mean (SD)]	3.04 (1.94)	4.17 (2.10)	**<0.001**	1.31 (1.15, 1.50)	3.45 (2.04)	5.07 (1.90)	**<0.001**	1.47 (1.25, 1.71)
Ever told by health professional had manic–depressive illness or bipolar	17 (18.3%)	33 (18.4%)	0.975	1.01 (0.53, 1.93)	41 (18.7%)	9 (16.7%)	0.727	0.88 (0.39, 1.92)
*Gender norms [mean (SD)]*								
Attitudes to gender relations	47.05 (7.25)	44.24 (7.11)	**0.002**	0.95 (0.91, 0.98)	45.08 (7.46)	44.38 (6.37)	0.343	0.98 (0.94, 1.02)
Attitudes to gender roles	28.57 (1.89)	27.58 (2.53)	**0.001**	0.81 (0.72, 0.92)	27.97 (2.38)	27.71 (2.35)	0.473	0.96 (0.85, 1.08)

England	Controlling behaviours				Technology facilitated abuse			
	No, *N* = 80	Yes, *N* = 143	*P*	OR (95% CI)	No, *N* = 150	Yes, *N* = 73	*P*	OR (95% CI)
*Demographics*								
Age [mean (SD)]	44.14 (9.08)	41.59 (9.78)	0.057	0.97 (0.94, 1.00)	45.06 (11.31)	36.65 (8.87)	**<0.001**	0.29 (0.13, 0.64)
Heterosexual	76 (96.2%)	138 (96.5%)	0.908	1.09 (0.25, 2.40)	146 (98.0%)	68 (93.2%)	0.069	0.28 (0.06, 1.20)
Live in the country of birth	73 (91.3%)	119 (83.2%)	0.096	0.47 (0.19, 1.16)	129 (86.0%)	63 (86.3%)	0.951	1.03 (0.46, 2.31)
No/primary schooling/left school without qualifications	30 (35.5%)	46 (32.2%)	0.420	0.79 (0.45, 2.43)	51 (34.0%)	25 (34.2%)	0.971	1.01 (0.56, 1.85)
Unemployed/receiving benefits	68 (85.0%)	125 (87.4%)	0.613	1.22 (0.56, 2.69)	127 (84.7%)	66 (90.4%)	0.238	1.71 (0.69, 4.19)
Homeless	20 (25.0%)	39 (27.3%)	0.712	1.12 (0.60, 2.10)	41 (27.3%)	18 (24.7%)	0.671	0.87 (0.46, 1.65)
*Intimate relationship*								
Infidelity	12 (15.6%)	38 (26.6%)	0.064	1.96 (0.95, 4.02)	28 (19.0%)	22 (30.1%)	0.065	1.83 (0.96, 3.50)
Believed that current/most recent partner has/had a problem with alcohol or drug use	38 (47.5%)	65 (45.5%)	0.769	0.92 (0.53, 1.59)	66 (44.00%)	37 (50.7%)	0.347	1.31 (0.75, 2.29)
*Substance use*								
In treatment for alcohol	28 (35.4%)	50 (35.0%)	0.943	0.98 (0.55, 1.74)	49 (32.9%)	29 (39.7%)	0.316	1.34 (0.75, 2.40)
In treatment for drug	62 (77.5%)	107 (74.08%)	0.655	0.86 (0.45, 1.65)	115 (76.7%)	54 (74.0%)	0.659	0.86 (0.45, 1.65)
Hazardous drinking	51 (63.8%)	94 (66.2%)	0.713	1.14 (0.63, 1.98)	95 (63.8%)	50 (68.5%)	0.486	1.24 (0.68, 2.43)
AUDIT total score	17.45 (14.13)	17.47 (13.35)	0.991	1.00 (0.98, 1.02)	16.96 (13.75)	18.49 (13.34)	0.431	1.01 (0.99, 1.03)
*Anger [mean (SD)]*								
Anger expression index	32.57 (14.45)	38.12 (14.21)	**0.006**	1.03 (1.01, 1.05)	33.75 (13.81)	40.98 (14.81)	**<0.001**	1.04 (1.01, 1.06)
*Violence*								
Victim controlling behaviours	52 (65.8%)	127 (88.8%)	**<0.001**	4.12 (2.05, 8.28)	110 (73.8%)	69 (94.5%)	**0.001**	6.12 (2.09, 17.87)
Victim TFA	27 (34.6%)	86 (60.6%)	**<0.001**	2.90 (1.63, 5.16)	55 (37.4%)	58 (79.5%)	**<0.001**	6.47 (3.35, 12.50)
IPV victimisation								
Emotional	46 (57.5%)	113 (80.1%)	**0.001**	2.98 (1.62, 5.47)	98 (66.2%)	61 (83.6%)	**0.016**	2.59 (1.28, 5.26)
Sexual	3 (3.8%)	16 (11.3%)	0.055	3.26 (0.92, 11.55)	10 (6.7%)	9 (12.3%)	0.160	1.95 (0.75, 5.04)
Moderate physical	7 (12.5%)	10 (9.7%)	0.586	0.75 (0.27, 2.10)	15 (14.7%)	2 (3.5%)	**0.028**	0.21 (0.05, 0.95)
Severe physical	49 (87.5%)	93 (90.3%)	0.586	1.33 (0.48, 3.71)	87 (85.3%)	55 (96.5%)	**0.028**	4.74 (1.04, 21.54)
IPV perpetration								
Emotional	42 (52.5%)	96 (67.6%)	**0.026**	1.89 (1.07, 3.31)	82 (55.0%)	56 (76.7%)	**0.002**	2.69 (1.43, 5.06)
Sexual	1 (1.3%)	10 (7.0%)	0.093	5.83 (0.73,46.45)	6 (4.1%)	5 (6.8%)	0.375	1.73 (0.51, 5.86)
Moderate physical	11 (36.8%)	18 (25.4%)	0.251	0.59 (0.23, 1.46)	20 (33.9%)	9 (21.4%)	0.172	0.53 (0.21, 1.32)
Severe physical	19 (63.3%)	53 (74.6%)	0.251	1.70 (0.68, 4.26)	39 (66.1%)	33 (78.6%)	0.172	1.88 (0.75, 4.69)
Ever physical fight with a man	74 (93.7%)	128 (89.5%)	0.300	0.58 (0.20, 1.65)	137 (91.9%)	65 (89.0%)	0.478	0.71 (0.28, 1.83)
*Mental health*								
PHQ total score [mean (SD)]	10.67 (7.54)	11.34 (6.43)	0.486	1.01 (0.97, 1.06)	10.78 (7.08)	11.78 (6.31)	0.313	1.02 (0.98, 1.06)
Probable depressive disorder	38 (47.5%)	73 (51.0%)	0.611	1.15 (0.67, 1.99)	74 (49.3%)	37 (50.7%)	0.850	1.06 (0.60, 1.85)
Number of adverse childhood experiences [mean (SD)]	3.57 (2.18)	4.57 (2.20)	**0.002**	1.23 (1.07, 1.41)	3.79 (2.22)	5.03 (2.05)	**<0.001**	1.30 (1.12, 1.50)
Ever told by health professional had manic‐depressive illness or bipolar	8 (10.0%)	28 (19.9%)	0.056	2.23 (0.96, 5.16)	22 (14.8%)	14 (19.4%)	0.377	1.39 (0.66, 2.92)
*Gender norms [mean (SD)]*								
Attitudes to gender relations	47.05 (7.25)	44.24 (7.11)	0.597	1.01 (0.96, 1.06)	48.41 (5.40)	49.23 (5.90)	0.300	1.03 (0.98, 1.08)
Attitudes to gender roles	28.57 (1.89)	27.57 (2.53)	0.086	0.88 (0.77, 1.02)	28.93 (2.48)	29.29 (1.77)	0.277	1.08 (0.94, 1.23)

ACE, adverse childhood experiences; AUDIT, Alcohol Use Disorders Identification Test; CI, confidence interval; IPV, intimate partner violence; OR, odds ratio; PHQ, Primary Care Evaluation of Mental Disorders Patient Health Questionnaire; TFA, technology facilitated abuse.

**Table 3:** Multivariate factors associated with the perpetration of controlling behaviours and technology facilitated abuse by country (excluding intimate partner violence victimisation)

	Controlling behaviours		Technology facilitated abuse	
	Brazil^a^	England^b^	Brazil^c^	England^d^
Severe physical IPV perpetration	3.29 (1.05, 10.34)	—	—	—
Number of adverse childhood experiences	1.45 (1.13, 1.86)	1.23 (1.06, 1.42)	—	—
Anger expression index	—	1.04 (1.01, 1.06)	—	—
Infidelity	—	1.97 (0.89, 4.37)	—	—
Age	—	—	0.88 (0.83, 0.94)	0.88 (0.82, 0.95)
*R* ^2^	0.21	0.17	0.35	0.25
Model fit	0.670	0.414	0.719	0.286

Nagelkerke *R* square was used to test *R* ^2^ and Hosmer and Lemeshow test to check the goodness of the model fit.

a Variables entered: Age, unemployed/receiving benefits, infidelity, in treatment for alcohol use, in treatment for drug use, AUDIT total score, anger expression index, ever physical fight with a man, probable depressive disorder, number of adverse childhood experiences, attitudes towards gender relations and attitudes towards gender roles, emotional IPV perpetration, severe physical IPV perpetration.

b Variables entered: Age, infidelity, live in country of birth, anger expression index, number of adverse childhood experiences, attitudes towards gender roles, emotional IPV perpetration.

c Variables entered: Age, sexuality, No/Primary schooling/Left school without qualifications, infidelity, current partner had problem with alcohol/drugs, in treatment for alcohol use, in treatment for drug use, anger expression index, probable depressive disorder, number of adverse childhood experiences, emotional IPV perpetration, severe physical IPV perpetration, sexual IPV perpetration.

d Variables entered: Age, sexuality, infidelity, anger expression index, number of adverse childhood experiences, emotional IPV perpetration, severe physical IPV perpetration.

AUDIT, Alcohol Use Disorders Identification Test; IPV, intimate partner violence.

**Table 4:** Multivariate factors associated with the perpetration of controlling behaviours and technology facilitated abuse by country (including intimate partner violence victimisation and/or perpetration)

	Controlling behaviours		Technology facilitated abuse (TFA)	
	Brazil^a^	England^b^	Brazil^c^	England^d^
Severe physical IPV perpetration	2.95 (0.92, 9.52)	—	—	—
Emotional IPV victimisation	—	2.68 (1.30, 5.52)	—	—
Number of adverse childhood experiences	1.40 (1.08, 1.80)	1.23 (1.04, 1.44)	—	—
Victim of controlling behaviour	4.17 (0.99, 17.57)	3.65 (1.63, 8.17)	—	—
Victim of TFA	—	—	3.39 (1.03, 11.15)	5.66 (1.64, 19.53)
Anger expression index	—	1.03 (1.01, 1.06)	—	—
Age	—	—	0.90 (0.84, 0.96)	0.88 (0.81, 0.96)
*R* ^2^	0.26	0.27	0.39	0.44
Model fit	0.478	0.354	0.792	0.767

Nagelkerke *R* square was used to test *R* ^2^ and Hosmer and Lemeshow test to check the goodness of the model fit.

a Variables entered: Age, unemployed/receiving benefits, infidelity, in treatment for alcohol use, in treatment for drug use, AUDIT total score, anger expression index, ever physical fight with a man, probable depressive disorder, number of adverse childhood experiences, attitudes towards gender relations, attitudes towards gender roles, emotional IPV victimisation, victim of controlling behaviour, victim of technology facilitated stalking, emotional IPV perpetration, severe physical IPV perpetration.

b Variables entered: Age, infidelity, live in the country of birth, anger expression index, number of adverse childhood experiences, attitudes towards gender roles, emotional IPV victimisation, victim of controlling behaviour, victim of technology facilitated stalking, emotional IPV perpetration.

c Variables entered: Age, heterosexual, No/Primary schooling/Left school without qualifications, infidelity, anger expression index, current partner had problem with alcohol/drugs, in treatment for alcohol use, in treatment for drug use, number of adverse childhood experiences, probable depressive disorder, emotional IPV victimisation, sexual IPV victimisation, victim of controlling behaviour, victim of technology facilitated stalking, emotional IPV perpetration, sexual IPV perpetration, severe physical IPV perpetration.

d Variables entered: Age, heterosexual, infidelity, anger expression index, number of adverse childhood experiences, emotional IPV victimisation, severe physical IPV victimisation, victim of controlling behaviour, victim of technology facilitated stalking, emotional IPV perpetration, severe physical IPV perpetration.

AUDIT, Alcohol Use Disorders Identification Test; IPV, intimate partner violence; TFA, technology facilitated abuse.

## Results

The sample characteristics and differences between countries are described elsewhere in this issue 33. Briefly, participants were aged 43 years (SD 10.6) on average, 96.6% were heterosexual (96.8% Brazil, 96.4% England), 14.1% were homeless (4.3% Brazil, 26.5% England), 50.6% had no/primary schooling only/left high school with no qualifications (63.9% Brazil, 34.1% England) and 93.3% lived in their country of birth (98.9% Brazil, 86.1% England). The most commonly used drugs in the past 30 days were cocaine (Brazil), crack, heroin (England) and cannabis; 70.2% reported hazardous drinking (74.0% Brazil, 65.3% England).

## Prevalence of controlling behaviour and TFA by country (Table 1)

Reported acts of controlling behaviour, including sexual jealousy towards current/most recent partner, were highly prevalent in both the Brazil (184/280, 65%) and English (143/64.1%) samples. The most common form of controlling behaviour reported in both countries was frequent thoughts that their partner was being unfaithful (169/519, 32.6%). Compared to participants from England, those from Brazil were more likely to have reported trying to isolate their partner from friends, to have tried to restrict their partners' contact with family, to get angry when their partner spoke to another man or have expected their partner to ask permission before seeking health care. Participants from England were more likely to report having ignored or treated their partners indifferently than participants from Brazil.

Participants from England (73/223, 32.7%) were almost twice as likely as participants from Brazil (57/280, 20.4%) to report TFA (OR 1.90, 95% CI 1.27, 2.85). Compared to participants from Brazil, those from England were more likely to have used text messages, phone, etc. to harass, threat or humiliate their partner.

### Variables associated with perpetrating controlling behaviour by country (Table 2)

Univariate analysis revealed that participants from Brazil who had perpetrated controlling behaviours were more likely than those who had not to be younger, to have been unfaithful to their current/most recent partner, to report unemployment/receiving benefits, receiving treatment for drug use, to have experienced victimisation and/or perpetration of emotional IPV, victimisation of sexual IPV, controlling behaviours and TFA from a partner, perpetrated severe physical IPV, have probable depressive disorder, report higher anger expression, report a greater number of ACE, to have had ever had a physical fight with another man and were more likely to hold gender stereotyped attitudes. Participants from Brazil who reported severe physical IPV perpetration were four times more likely to report controlling behaviours towards their current/most recent partner (OR 4.00, 95% CI 1.45, 11.06). Participants from Brazil who had not perpetrated any controlling behaviours were more likely to support gender equitable norms than those who had perpetrated controlling behaviours.

Participants from England who had perpetrated controlling behaviours were more likely than those who had not to report lifetime victimisation and/or perpetration of emotional IPV, to have experienced controlling behaviours and TFA from a partner, report higher anger expression and have experienced a greater number of adverse childhood experiences.

### Variables associated with TFA by country (Table 2)

The perpetration of TFA was strongly associated with other forms of controlling behaviours. Participants who reported perpetrating controlling behaviours were almost seven times more likely to also have reported perpetrating TFA (OR 6.94, 95% CI 3.7, 12.76). TFA perpetration was also associated with other types of IPV perpetration.

For participants from Brazil, the odds of perpetrating TFA were greater for those who were younger, reported infidelity, were receiving treatment for drug use (rather than alcohol use only), reported higher anger expression, reported higher depressive symptomatology and a greater number of ACE, reported emotional and/or sexual IPV perpetration, who had had experienced emotional or sexual IPV victimisation, controlling behaviours and TFA from a partner. While moderate physical IPV (e.g. pushing, slapping) perpetration in the Brazil cohort was associated with a decrease in the likelihood of perpetrating TFA, participants who had perpetrated severe physical IPV were over nine times more likely to report TFA (OR 9.03, 95% CI 1.15, 70.68).

Participants from England who reported perpetrating emotional IPV were over twice as likely to report TFA (OR 2.69, 95% CI 1.43, 5.06). In addition participants from England who had perpetrated TFA were younger, had experienced a greater number of ACE, expressed higher anger, had experienced emotional or severe IPV victimisation, controlling behaviours and TFA from a partner.

### Risk factors for controlling behaviours and TFA by country

Excluding IPV victimisation from the multivariate models; the perpetration of controlling behaviours was associated with a higher number of ACE, higher anger expression (England) and severe physical IPV perpetration (Brazil), and TFA was associated with younger age (Table 3). Including both IPV victimisation and IPV perpetration in the multivariate models; the perpetration of controlling behaviour was associated with experiencing a higher number of ACE, higher anger expression (England), emotional IPV victimisation (England) and experiencing controlling behaviour from a partner (England, marginally significant Brazil) (Table 4). Severe physical IPV perpetration and experiencing controlling behaviour from a partner were marginally significant in this model for Brazil. The perpetration of TFA was associated with younger age (when IPV victimisation was and was not included in the model) and experiencing TFA (when IPV victimisation was included in the model) from a partner in both England and Brazil.

## Discussion

A significant proportion of men receiving treatment for substance use had perpetrated controlling behaviours (64% in England and 65% in Brazil) and TFA (33% in England and 20% in Brazil) towards their current/most recent partner. While we could find no studies among similar populations to compare these rates to, a recent study of 42 000 women across the 28 Member States of the European Union suggests that the prevalence of such behaviours may be higher among men receiving treatment for substance use 58. While 35% and 5% had experienced controlling behaviours or TFA, respectively, women who reported their partners got drunk at least once a month reported experiencing greater psychological abuse. Similar to our findings, younger women in that study were more likely to experience TFA than older women.

Although the prevalence of TFA in our sample was lower than the perpetration of controlling behaviours, other studies suggest that TFA is an emerging form of IPV perpetration 19, 59, 60. Moreover, TFA may be another form of controlling behaviour as the odds of perpetrating TFA increased almost seven fold with the perpetration of controlling behaviours. To our knowledge, the current study is the first to report the association between controlling behaviour and TFA. Similar rates of perpetrating controlling behaviours between countries were reported, but lower rates of perpetrating TFA were reported in Brazil compared to England, potentially because of lower access to such technologies in Brazil. In 2015, 76% of the UK adult population had access to the internet 61, and 52% of people in treatment for substance use had smartphones 62 compared to 56% of the Brazilian population having access to the internet and 28% having a smartphone 63, illustrating the role that widespread access to internet and smartphones has on providing additional opportunities for perpetrators to control, stalk and abuse their partners 19, 22.

In univariate analysis, other forms of IPV perpetration were also found to be associated with controlling behaviours and TFA. In England, perpetration of controlling behaviours and TFA were only associated with the perpetration of emotional IPV. In Brazil, perpetrating controlling behaviours and TFA were associated with severe physical and sexual IPV perpetration. Research confirms the association between the perpetration of controlling behaviours and emotional, physical and sexual IPV 29, 38, 39, 64, and between cyberstalking (or TFA) and in‐person intimate partner psychological, physical and sexual abuse 23. Indeed a comparative analysis of population‐based data from 12 countries in Latin America and the Caribbean found that the percentage of women who reported at least three controlling behaviours by their partner was around two to three times greater among those who also reported physical or sexual IPV 64. In both England and Brazil, participants in our study who were more supportive of gender equitable norms were less likely to report controlling behaviour. It is interesting that in spite of the higher support for gender equitable relations and less gender stereotyped attitudes in England compared to Brazil 48, the prevalence of perpetrating controlling behaviour was similar. While Stark's 6 suggestion that physical IPV may be used to maintain ‘coercive control’ is partially supported in the Brazil data, the data from England suggests that more subtle forms of IPV, including TFA, may also be used to enforce control in a cultural context where male domination and IPV are less socially accepted.

The univariate analysis highlighted that in both countries men who perceived that their partner had used controlling behaviours or TFA, emotional, sexual (Brazil) or severe physical (England) violence against them were more likely to themselves perpetrate controlling behaviours or TFA towards their partner, suggesting that these behaviours may be perceived as mutual in the relationship. These findings highlight the need to include dyad accounts of IPV context and events to better understand the meanings and uses of these behaviours in different cultural contexts. The typology of intimate terrorism was partly supported in Brazil, when IPV victimisation was excluded, as men who reported such behaviour were also more likely to perpetrate severe physical IPV, whereas only ACE and high anger expression were significant in the model for England.

Our findings support cultural differences in variables associated with controlling behaviour and TFA that are difficult to interpret. It is also possible that differences in the profile of users of drug treatment services may have contributed. Excluding IPV victimisation from the multivariate models; controlling behaviours were associated with a higher number of ACE, higher anger expression (England) and severe physical IPV perpetration (Brazil), and TFA was associated with younger age. When IPV victimisation and perpetration were included in the multivariate model, further country differences were reported. Perpetrating controlling behaviour was associated with experiencing a higher number of ACE (England and Brazil), higher anger expression (England), emotional IPV victimisation (England) and experiencing controlling behaviour from a partner (England). Severe physical IPV perpetration and experiencing controlling behaviours from a partner were marginally significant in the model for Brazil. For both England and Brazil, younger age and experiencing TFA from a partner remained significant in the model predicting TFA, when IPV victimisation was included.

A 12‐country study of IPV suggests that severe physical violence is highly prevalent among women in Latin America and the Caribbean, with a majority of those who experienced any physical IPV also experiencing ‘severe’ acts of physical violence (e.g. being hit with a fist, threatened or wounded with a knife or other weapon) 64. It is possible that in cultures such as England where there is lower acceptability of severe physical IPV, controlling behaviour may substitute for physical IPV. Previous studies show that factors associated with IPV perpetration may vary across countries 38, possibly because of cultural differences in the status of women or acceptability of interpersonal violence 37.

Similar to our findings, there is an extensive literature showing that ACE and anger are influential factors for IPV perpetration 29, 65, 66. Studies suggest that experiencing ACE can have an impact on the threat‐appraisal response system and result in hyper‐reactivity to later stressors in life 65, 67, 68. Heightened reactivity to stress has been argued to be the potential link between childhood adversities, anger and IPV perpetration 65, 66, 69.

### Implications

There has been significant progress in acknowledging the severe impact of controlling behaviours in intimate relationships. In the UK, the *Serious Crime* *Act* (2015) established a new offence of controlling or coercive behaviour in intimate or familiar relationships, carrying a maximum sentence of five years' imprisonment, a fine of both 70. Although in Brazil there is no specific offence for controlling or coercive behaviours, the Maria da Penha law introduced in 2006 has been pivotal in punishing those who cause harm to their female partners, including from psychological abuse. There is, however, limited evidence to recommend risk assessment tools in predicting and measuring controlling behaviour 27, but TFA should be considered within any assessment of IPV. In addition, there is an urgent need for policy and legal responses to address TFA in the context of IPV. The fact that younger participants were more likely to perpetrate TFA suggests that the negative use of technology in intimate relationships should be a focus of prevention strategies to reduce IPV perpetration. Recent research highlights that three out of four stalking cases (including TFA) were not reported to the Police. This could be the result of a lack of understanding about whether TFA constitutes IPV or that victims minimise TFA experiences. Behaviour change approaches aimed at tackling IPV should consider challenging existing norms, attitudes and beliefs that reinforce men's violence and conflict resolution 38, 71. There is a need for IPV prevention or intervention programmes to address violence and abuse perpetrated in person as well as through technology, especially among younger perpetrators. Our findings concerning the risk factors for perpetrating controlling behaviours and TFA provide additional information to support delivering interventions to address IPV among male perpetrators who receive treatment for substance use.

### Limitations

The cross‐sectional nature of the study means that causality cannot be inferred. Questions used to assess controlling behaviours and TFA were measured by the occurrence of a single event rather than occurrence and frequency together. Therefore, prevalence of these behaviours may be over‐reported. Furthermore, participant reports of perpetration and victimisation were not corroborated by their current or ex‐partners. Results should be interpreted with caution where wide confidence intervals were reported. Despite these limitations, this study was the first to examine controlling behaviours and TFA among a large cross‐cultural sample of men receiving treatment for substance use. Unfortunately, because of the nature of the sample, it was not possible to make inferences about the role of drugs and alcohol in this study.

## Conclusion

Understanding different forms of IPV is central to understanding how and why IPV prevails 13. This study provides evidence that controlling behaviour is common among men in substance use treatment and is associated with other forms of IPV. Technological advancements provide perpetrators with additional opportunities to control their partners, especially among the younger population. Risk factors for perpetrating controlling behaviours and TFA were identified that could offer ways of improving interventions aimed at preventing and reducing IPV.
